# Production Task Allocation Decision Based on Cloud Robot Cell-Line

**DOI:** 10.1155/2022/5892943

**Published:** 2022-03-24

**Authors:** Yi Du, Yongqiang Wang, Jialin Wang, Tianpeng Zhang

**Affiliations:** ^1^Anyang Institute of Technology School of Electronic Information and Electrical Engineering, Anyang 455000, China; ^2^Anyang Branch, China Mobile Tietong, Anyang 455000, China; ^3^Hangzhou Normal University, Hangzhou 310000, China

## Abstract

Cloud manufacturing is a new service-oriented efficient and low-consumption agile manufacturing mode integrating information, manufacturing, Internet of Things, and other technologies. One of the key decisions of production enterprises is production task allocation based on cloud robot cell-line, which determines the efficiency and flexibility of the production system and affects various production links, such as job-shop logistics, production planning, and production scheduling. This paper explores the production task allocation, from the angle of the optimal combination of cloud manufacturing resources. First, a mathematical model was established, based on the transport cost of different sub-tasks and the tardiness cost of product delivery, and solved by quantum firefly algorithm (QFA). Next, QFA was proved superior to traditional firefly algorithm (FA), improved FA, and the FA optimized by cat swarm optimization (CSO-FA), in terms of time complexity and spatial complexity. The research enriches the theory and methodology of allocating operation-level cloud manufacturing resources based on cloud robot cell-line and provides decision support to manufacturers, which want to implement operation-level allocation of cloud manufacturing resources based on cloud robot cell-line.

## 1. Introduction

Big manufacturers like China are improving the intelligence level of the manufacturing industry. Cloud intelligence can increase the utilization efficiency of production lines, lower the production cost, and avoid repeated investment and construction, contributing to the improvement of our living standards.

Unlike traditional flow lines, the cell-line combines a few similar stations into a novel type of production line. There are two advantages of the cell-line: First, it is of the same production efficiency as a conveyor belt flow line. Second, the flexible production of production units adapts well to the market needs of various small batches of products. Therefore, the cell-line is regarded as an ideal production mode to realize massive customization. Besides, the cell-line provides a green and economic production method.

Scholars at home and abroad have explored robotic automation production lines extensively. Focusing on the variable process pathway, Tian et al. [1] established a minimum delay time function and constructed a scheduling module, in the light of collaborative, differentiated agents. In addition, agents were adopted to realize global optimization, aiming to minimize the makespan. Zacharia and Nearchou [2] summarized the features of job-shop scheduling with multiple robotic manufacturing units, created a mathematical model for minimizing the makespan of the problem, and solved the model effectively with a hybrid genetic algorithm. Liu et al. [3] utilized the minimum completion time to solve the flexible job-shop scheduling with loading/unloading robots, established a mathematical model of the problem, and searched for the scheduling results with a tabu search and greedy algorithm. Miyake [4] used the minimum production cycle to solve the multi-process job-shop scheduling with transport time, constructed a nonlinear mathematical model, and solved the model with improved genetic algorithm, realizing batch scheduling. Du et al. [5] explored the reconstruction and scheduling of multi-stage variable-batch production lines in labor-intensive enterprises, adopted different algorithms for production lines of different scales, and compared the applicable scopes of two joint optimization algorithms with examples. Wu et al. [6] studied the optimal control of event-driven conveyor belt feeding and processing system.

There are some limitations that present opportunities for future research. Our Stackelberg game modeled the operator as the leader and the suppliers as the followers. It might be beneficial for practical applications to explore other power structures. In future research, a supplier-led power structure (e.g., a large-scale enterprise building a platform) could be an important extension of this model. Moreover, we studied two types of substitutable cloud services that can satisfy client demands. In reality, there might be multiple types of cloud services that meet the same requirement. Future research should consider the case of multiple types of substitutable cloud services in the CMfg system.

Cao et al. [7] stated that there are some limitations that present opportunities for future research. Our Stackelberg game modeled the operator as the leader and the suppliers as the followers. It might be beneficial for practical applications to explore other power structures. In future research, a supplier-led power structure (e.g., a large-scale enterprise building a platform) could be an important extension of this model. Moreover, we studied two types of substitutable cloud services that can satisfy client demands. In reality, there might be multiple types of cloud services that meet the same requirement. Future research should consider the case of multiple types of substitutable cloud services in the CMfg system. Carlucci et al. [8] observed that a key limitation of the research is the use of homogeneous resources shared in the network. Future research could take into account different categories of resources that are characterized by different rules in order to determine the win or lose state. Another important research issue to further investigate is the dynamic composition of the network based on the obtained performance, i.e., how the participant in the network can decide to enter or leave the network.

To sum up, there are very few studies on cell-line cloud manufacturing resource scheduling based on cloud robots, which could avoid resource waste, repetitive building of production lines, and low utilization of units. This paper explores production task allocation from the angle of the optimal combination of cloud manufacturing resources. Specifically, a mathematical model was established based on the transport cost of different sub-tasks and the tardiness cost of product delivery, and solved by quantum firefly algorithm (QFA). The effectiveness of the model and algorithm was demonstrated through example analysis.

At present, there are many researches on the single technology of cloud manufacturing. With the in-depth research and application of cloud manufacturing, the cloud manufacturing service platform oriented to the whole life cycle of products will become the next research hotspot. New product development is an important life cycle stage of complex electronic products. There are very few researches on cloud manufacturing resource scheduling of the Japanese unit production line based on cloud robot. However, the results of researches can avoid the waste of resources, repeated line construction, and low utilization rate of units.

The driving effect of electronic information industry to China's economic growth is showing a sustained and scale of growth. Moreover, the promotion and penetration effect to the upgrading of traditional industrial structure gradually increases. The high-tech industry represented by the electronic information industry has developed far faster than the traditional industry, and the export of high-tech products has shown a rapid growth trend. By observing the development of electronic information industry and the track of technological upgrading, it is concluded that China has a strong and obvious comparative advantage in processing and assembly, and the manufacturing of electronic information products in the world is forming a tendency to be concentrated in some areas of China. First, due to the epidemic, foreign orders have increased, and some manufacturers have blindly expanded production scale, resulting in serious excess production capacity. However, some manufacturers have insufficient orders and idle production resources. Second, the processing technology of small batch products is more and more complex and often the production resources of an enterprise cannot meet the requirements of product processing. The cloud platform can contact enterprises in multiple regions to complete manufacturing resource scheduling tasks together.

## 2. Problem Formulation

In 2010, James first put forward the definition of “cloud” robot; it points out that the cloud robotics is robot to store and retrieve information, such as a robot with a camera around the photo upload cloud server, a search on the server, find the similar photos, to plan the robot path tracing, and store the pictures, as search information for the back of the machine. All robots can share this library to reduce the developer effort and time.

Suppose there are requests for *I* types of products on the cloud platform. Each product request contains N sub-tasks *F*_*i*_, *i* = {1, 2,…, *N*} to be processed on *k* intelligent cloud scheduling production lines. Each sub-task contains *P*_*i*_ operations, and *K*_*i*_ optional production lines. The production task can be expressed as follows:

(1) $$\begin{array}{c} \text{F}={\text{min}}_{\left(f_{1}f_{2}\right)}, \end{array}$$

where *f*_1_ is the minimum processing time of the manufacturing resources of a task, which depends on the sub-task with the longest processing time:

(2) $$\begin{array}{c} f_{1}=\text{min}\left(\text{max}\overset{T}{\underset{t=1}{\sum }}\overset{I}{\underset{i=1}{\sum }}\overset{K}{\underset{k=1}{\sum }}\left({\text{e}}_{\text{tik}}\varepsilon_{\text{ti}}{\text{L}}_{\text{tik}}+x_{k,k+i}q_{tik}h_{ti}\right)\right), \end{array}$$

where *e*_*ikt*_ is a 0–1 variable (if *e*_*ikt*_ = 1, product *i* is processed through *t* stages on production line *k*; if *e*_*ikt*_ = 0, product *i* is not processed through *t* stages on production line k); *ε*_*ti*_ is the number of product *i* through *t* stages; *l*_*ikt*_ is the processing time of product *i* through *t* stages on production line *k*; *q*_*tik*_ is the transport time of 1,000 products *i* from production line *k* to production line *k*+1; *x*_*k*,*k*_ _*+*_ _*i*_ is a 0–1 variable (if *x*_*k*,*k*_ _*+*_ _*i*_ = 1, product *i* is transported from production line *k* to production line *k*+1 through *t* stages; if *x*_*k*,*k*_ _*+*_ _*i*_ = 0, product *i* is not transported from production line *k* to production line *k*+1 through *t* stages); *h*_*ti*_ is the multiple of 1,000 products *i* through *t* stages.

*f*_2_ is the minimum cost of all sub-tasks of manufacturing resource processing:

(3) $$\begin{array}{c} f_{2}=\text{min}\left(\text{max}\overset{T}{\underset{t=1}{\sum }}\overset{I}{\underset{i=1}{\sum }}\overset{K}{\underset{k=1}{\sum }}\left({\text{e}}_{\text{tik}}\varepsilon_{\text{ti}}{\text{C}}_{\text{tik}}+\left(f_{itk}-s_{itk}\right)*d_{it}\right)\right), \end{array}$$

where Ctik is the manufacturing cost of product *i* through *t* stages on production line *k*; fitk is the processing time of product *i* through *t* stages on production line *k* (including transport time); sitk is the delivery period of product *i* through *t* stages on production line *k*; and ditfitk is the tardiness cost constraint of product *i* through *t* stages.

The processing of the current operation of a sub-task should not begin before the completion of the previous operation:

(4) $$\begin{array}{c} \overset{M_{ij}}{\underset{k=1}{\sum }}e_{ijkt}=1, \end{array}$$

where *t*_end_*ij*__ and *t*_start_*i*(*j*+1)__ are the completion time of the previous operation and the start time of the current operation, respectively.

Operation is the minimum granularity of scheduling. Each operation can only occupy one logic manufacturing resource.

## 3. Algorithm Design

The QFA is designed in the following steps [9–14]. First, a swarm of *h* quantum fireflies is generated. Each quantum firefly has a quantum position and a position. The position of each quantum firefly is represented by a D-dimensional numerical string {0, 1}, with *D* being the dimensionality of the solution space. In the t-th iteration, the quantum position of the i-th quantum firefly can be expressed as follows:

(5) $$\begin{array}{c} v_{i}^{t}=\left[\begin{array}{ccc} \alpha_{i1}^{t} & \alpha_{i2}^{t}... & \alpha_{i1\;D}^{t}\\ \beta_{i1}^{t} & \beta_{i2}^{t}... & \beta_{i\;D}^{t} \end{array}\right], \end{array}$$

where (*α*_*il*_^*t*^)^2^+(*β*_*il*_^*t*^)^2^=1(1=1,2,…, *D*). The quantum positions *α*_*il*_^*t*^ and *β*_*il*_^*t*^ satisfy 0 ≤*α*_*il*_^*t*^ ≤ 1, and 0 ≤*β*_*il*_^*t*^ ≤ 1. At the beginning, all the quantum positions of the quantum fireflies are initialized as 1. For simplicity, the quantum position of the i-th quantum firefly in the t-th iteration can be denoted as *v*_*i*_^*t*^=(*v*_*i*1_^*t*^, *v*_*i*2_^*t*^,…, *v*_*iD*_^*t*^) (*i* = 1, 2, ..., *h*). The position of a quantum firefly can be derived from the measured quantum position. The position of the i-th quantum firefly can be expressed as *x*_*i*_^*t*^=(*x*_*i*1_^*t*^, *x*_*i*2_^*t*^.…*x*_*iD*_^*t*^) (*i* = 1, 2, ..., *h*). The global optimal position of the i-th quantum firefly in the t-th iteration can be expressed as *p*_*g*_^*t*^=(*p*_*g*1_^*t*^, *p*_*g*2_^*t*^,…, *p*_*gD*_^*t*^).

According to the fluorescein update rule, the fitness F (*p*_*i*_^*t*^) of quantum firefly *i* (*i* = 1, 2, ..., *h*) at the local optimal position in the t-th iteration can be converted into fluorescein *L*_*i*_(*t*):

(6) $$\begin{array}{c} L_{i}\left(t\right)=\left(1-\gamma \right)L_{i}\left(t-1\right)+\varepsilon F\left(p_{i}^{t}\right), \end{array}$$

where *γ* ∈ [0,1] is the disappearance rate of fluorescein, which gradually weakens with the growing distance and media absorption; *ε* is the update rate of fluorescein. The i-th quantum firefly obtains the learning neighborhood by certain rules. The quantum fireflies are selected from the neighborhood based on the fluorescein value and position similarity. In the t-th iteration, the i-th quantum firefly obtains the learning neighborhood by the following equation:

(7) $$\begin{array}{c} N_{i}\left(t\right)=\left\{q|D-d_{iq}\left(t\right)\leq r_{i}\left(t\right)且L_{i}\left(t\right)\leq L_{q}\left(t\right)\right\}, \end{array}$$

where *N*_*i*_(*t*) is the set of labels of the learning neighborhood of the i-th quantum firefly; *d*_*iq*_(*t*) is the distance between the local optimal positions of the i-th and q-th quantum fireflies; *r*_*i*_(*t*) is the radius of the dynamic decision domain of the i-th quantum firefly; and *L*_*q*_(*t*) is the fluorescein value of the q-th quantum firefly in the t-th iteration. The number of quantum fireflies in the learning neighborhood equals the number of labels in the label set of that neighborhood in the current iteration.

In each iteration, each quantum firefly selects its moving direction according to the quantum fireflies in the neighborhood. In the t-th iteration, the probability for the i-th quantum firefly to move to the q-th quantum firefly in its neighborhood can be expressed as

(8) $$\begin{array}{c} P_{iq}^{\prime }=\frac{L_{q}\left(t\right)-L_{i}\left(t\right)}{\sum_{j=N_{i}\left(t\right)}\left[L_{j}\left(t\right)-L_{i}\left(t\right)\right]}. \end{array}$$

In each iteration, if the learning neighborhood of the i-th quantum firefly is empty, then the evolution of the l-th dimensional quantum position of that quantum firefly can be expressed as follows:

(9) $$\begin{array}{c} v_{il}^{t+1}=\left\{\begin{array}{c} Nv_{il}^{t}\;\theta_{il}^{t+1}=0\;an\;d\;\mu_{il}^{t+1}<c_{1}\\ abs\left[U\left(\theta_{il}^{t+1}\right)v_{il}^{t}\right] \end{array}\right), \end{array}$$

where quantum rotation angle *θ*_*il*_^*t*+1^=*e*_1_(*p*_*il*_^*t*^ − *x*_*il*_^*t*^)+*e*_2_(*p*_*gl*_^*t*^ − *x*_*il*_^*t*^)*i*=1,2, ..., *h*; l=1,2, ..., *D*; *e*_1_ and *e*_2_ are the degree of influence of local optimal position and global optimal position on quantum rotation angle, respectively; *μ*_*il*_^*t*+1^ is a random number uniformly distributed in [0, 1]; *c*_1_, a constant in [0, 1/*D*], is the mutation probability of the quantum position at the quantum rotation angle of 0; *abs*(.) is the absolute value that limits each dimension of the quantum position in [0, 1]; *U*(*θ*_*il*_^*t*+1^)  =  $\left[\begin{array}{c} cos\theta_{il}^{t+1}\\ sin\theta_{il}^{t+1} \end{array}\begin{array}{c} -sin\theta_{il}^{t+1}\\ cos\theta_{il}^{t+1} \end{array}\right]$ is the quantum rotation gate; and $\bar{N}=\left[\begin{array}{cc} 0 & 1\\ 1 & 0 \end{array}\right]=\left[\begin{array}{cc} 0 & 1\\ 1 & 0 \end{array}\right]$ is the quantum NOT gate.

If the learning neighborhood of the i-th quantum firefly is not empty, each quantum firefly labeled *z* in the neighborhood satisfies *z* =  $\text{arg }{\text{max}}_{\text{q}\in {\text{N}}_{\text{it}}}^{\text{max}}\left\{\bar{{\text{P}}_{\text{iq}}}\left(\text{t}\right)\right\}$. In the t-th iteration, then the evolution of the l-th dimensional quantum position of the i-th quantum firefly can be expressed as follows:

(10) $$\begin{array}{c} v_{il}^{t+1}=\left\{\begin{array}{c} \bar{N}v_{il}^{t}\;\theta_{il}^{t+1}=0\;an\;d\;\mu_{il}^{t+1}<c_{2}\\ abs\left[U\left(\theta_{il}^{t+1}\right)v_{il}^{t}\right] \end{array}\right), \end{array}$$

where quantum rotation angle *θ*_*il*_^*t*+1^=*e*_3_(*p*_*il*_^*t*^ − *x*_*il*_^*t*^)+*e*_4_(*p*_*zl*_^*t*^ − *x*_*il*_^*t*^)++*e*_5_(*p*_*gl*_^*t*^ − *x*_*il*_^*t*^); i=1,2, ..., h; l=1,2, ..., D; *p*_*zl*_^*t*^ is the l-th dimension of the local optimal position, i.e., the position with the highest fluorescein, in the learning neighborhood of the i-th quantum firefly; *e*_3_, *e*_4_, and *e*_5_ are the degree of influence of the local optimal position of the i-th quantum firefly, the local optimal position, i.e., the position with the highest fluorescein, in the learning neighborhood of the i-th quantum firefly, and the global optimal position of the i-th quantum firefly on quantum rotation angle, respectively; *c*_2_, a constant in [0, 1/*D*], is the mutation probability of the quantum position at the quantum rotation angle of 0. The position of the i-th quantum firefly can be derived from the measured quantum position:

(11) $$\begin{array}{c} x_{il}^{t+1}=\left\{\begin{array}{c} 1,\eta_{il}^{t+1}>{\left(\alpha_{il}^{t+1}\right)}^{2}\\ 0,\eta_{il}^{t+1}\leq {\left(\alpha_{il}^{t+1}\right)}^{2} \end{array}\right), \end{array}$$

where *l* = 1,2, ..., D; *η*_*il*_^*t*+1^ ∈ [0,1] is a random number obeying uniform distribution; (*α*_*il*_^*t*+1^)^2^ is the probability of zero appearing at quantum position *v*_*il*_^*t*+1^.

The radius of the dynamic decision domain of the i-th quantum firefly can be updated by the following expression:

(12) $$\begin{array}{c} r_{i}\left(t+1\right)=\min\left\{R_{s},\text{max}\left[0,r_{i}\left(t\right)+\zeta \left\{n_{t}-size\left[N_{i}\left(t\right)\right]\right\}\right)\right\}, \end{array}$$

where *ζ* is a constant update rate of the dynamic decision domain; *R*_*s*_ ≥ *r*_*i*_(*t*) is a constant perception domain; min and max are the minimum and maximum functions, respectively; *n*_*t*_ is the parameter controlling the number of quantum fireflies in the learning neighborhood; and size[*N*_*i*_(*t*)] is the number of quantum fireflies in the learning neighborhood of the i-th quantum firefly.

For the cloud scheduling of production line, the fitness function of the QFA should minimize the production time of the production line. After computing the maximum production time and cost of cloud scheduling, the minimization of the two values is adopted as the optimization goal. Then, the fitness function of the current position *x*_*i*_^*t*^=(*x*_*i*1_^*t*^, *x*_*i*2_^*t*^,…, *x*_*i*  *D*_^*t*^) (*i* = 1,2, ..., h) of the i-th firefly can be expressed as follows:

(13) $$\begin{array}{c} F\left(x_{i}^{t}\right)=\left\{\begin{array}{c} -MSLL\left(x_{i}^{t}\right),IF\;crat\leq erat\\ -\rho .MSLL\left(x_{i}^{t}\right) \end{array}\right), \end{array}$$

where *MSLL*(*x*_*i*_^*t*^) is the maximum production time and cost of cloud scheduling constructed by *x*_*i*_^*t*^; *ρ* ≤ 1;  *crat* and *erat* are the calculated and expected coverage rates, respectively [15–19].

Based on the QFA, the production time and cost of the cloud manufacturing production line can be minimized through the following steps:

**Step 1 .**

Establish the mathematical model of the production time and cost of cloud computing production line, determine the key parameters of the QFA corresponding to the production time and cost, initialize the quantum positions of quantum fireflies, and measure their positions.

**Step 2 .**

Map the position of each quantum firefly to the corresponding parameters of the established model, import these parameters to the fitness function, compute the fitness at the position of the quantum firefly, and determine the local and global optimal positions according to the fitness.

**Step 3 .**

Update the fluorescein value and learning neighborhood of each quantum firefly, according to the fitness at the local optimal position of that quantum firefly.

**Step 4 .**

Change the quantum position of each quantum firefly with new data.

**Step 5 .**

Change the radius of the dynamic decision domain of each quantum firefly with new data.

**Step 6 .**

Compute the fitness of each firefly at the new position by the fitness function and determine the local and global optimal positions according to the fitness again.

**Step 7 .**

If the maximum number of iterations is reached, execute Step 8; otherwise, return to Step 3.

**Step 8 .**

**Figure 1:**
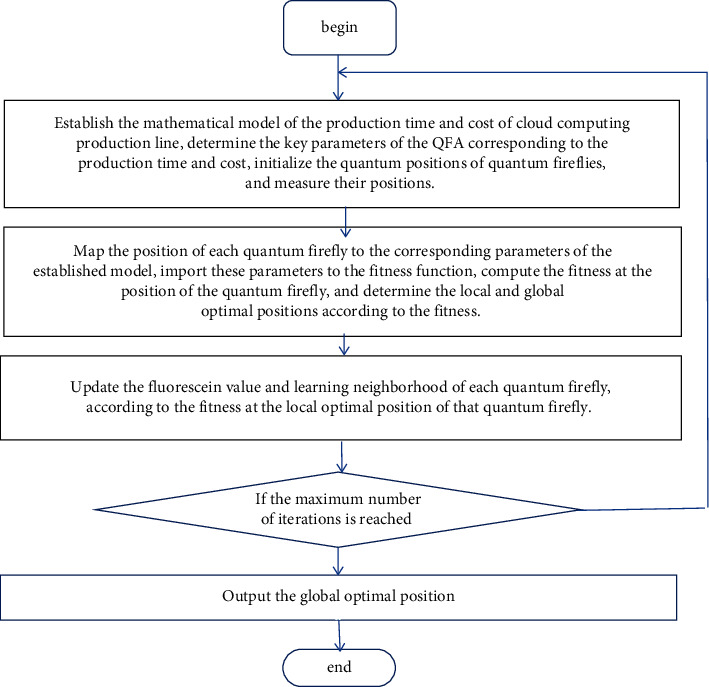
Program flow chart.

Output the global optimal position [20–25].(Flow chart as Figure 1)

The test example and calculation results are shown in Tables 1 and 2, respectively.

## 4. Example Analysis

Two swarms were initialized in our example. For convenience, each type of the product can be used after completion one production cycle. The initial swarms 1 and 2 were combinations of ones in each row of Table 1. Our algorithm IFA was compared with traditional firefly algorithm (FA), improved FA, and the FA optimized by cat swarm optimization (CSO-FA). The swarm size for all these intelligent optimization algorithms was fixed at 50(Set in the program). The maximum number of iterations was set to 350 (Set in the program), the expected coverage rate to 0.7(In (13), *erat* is the expected filling rate), *ρ*  = 0.001 (In Formula (13) *ρ* ≤ 1), the update rate of dynamic decision domain to 0.8 (Formula (12) *ζ* is the update rate of the dynamic decision domain), the initial fluorescein value to 5, *e*_1_=0.06*e*_2_=0.03*e*_3_=0.06*e*_4_=0.03, *e*_5_=0.01, *θ*_*il*_^*t*+1^=*e*_3_(*p*_*il*_^*t*^ − *x*_*il*_^*t*^)+*e*_4_(*p*_*zl*_^*t*^ − *x*_*il*_^*t*^)++*e*_5_(*p*_*gl*_^*t*^ − *x*_*il*_^*t*^); i=1,2, ..., h; l=1,2, ..., D; *p*_*zl*_^*t*^ and *e*_2_ are the degree of influence of local optimal position and global optimal position on quantum rotation angle, *θ*_*il*_^*t*+1^=*e*_3_(*p*_*il*_^*t*^ − *x*_*il*_^*t*^)+*e*_4_(*p*_*zl*_^*t*^ − *x*_*il*_^*t*^)++*e*_5_(*p*_*gl*_^*t*^ − *x*_*il*_^*t*^); i=1,2, ..., h; l=1,2, ..., D; *p*_*zl*_^*t*^ is the l-th dimension of the local optimal position, i.e., the position with the highest fluorescein, in the learning neighborhood of the i-th quantum firefly; *e*_3_, *e*_4_, and *e*_5_ are the degree of influence of the local optimal position of the i-th quantum firefly) and *c*_1_  =  *c*_2_  = 0.1/*D*.

## 5. Conclusions

**Figure 2:**
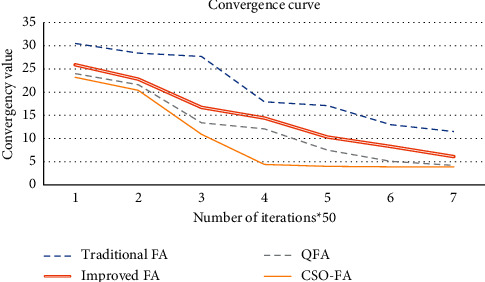
Convergence curves of different algorithms.

**Figure 3:**
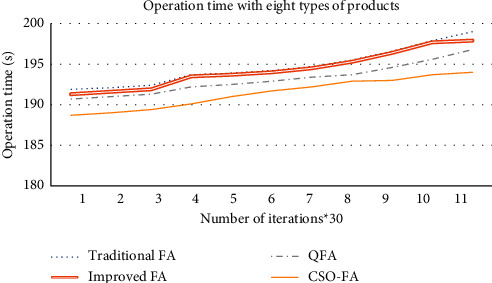
Operation time of different algorithms.

This paper mainly solves the cloud robot scheduling of a cell-line involving various small batches of products, which are produced in multiple cycles, in the light of transport cost. The QFA was adopted to reasonably allocate the resources in the cloud environment. Besides, the ideas of quantum computing were added to the FA to speed up the search, while maintaining the swarm search ability. Compared with the traditional FA, improved FA, and CSO-FA, the proposed QFA (as Figures 2 and 3) achieves excellent optimization ability and a fast search speed. Finally, our algorithm was demonstrated through an example analysis on the production order scheduling of an electronic product manufacturer. The results show that the QFA is capable of effectively solving the multiobjective scheduling of cloud manufacturing resources [26, 27].

## Figures and Tables

**Table 1:** Example of cloud manufacturing resource allocation.

Order	*d*_*i*_ (10,000 yuan/1,000 pieces/day)	Suborder	Production line	*c*_*ik*_ (10,000 yuan/1,000 pieces)	*l*_*ik*_/day
J_1_	1	*F*_1,1_	*K*_3_/ *K*_5_/ *K*_7_	3/3/5	5/5/3
		*F*_1,2_	*K*_1_/ *K*_6_/ *K*_7_	5/8/8	8/5/5
		*F*_1,3_	*K*_3_/ *K*_5_	6/3	3/6
		*F*_1,4_	*K*_5_/ *K*_9_	4/3	3/4

J_2_	1	*F*_2,1_	*K*_4_/ *K*_9_	10/4	4/10
		*F*_2,2_	*K*_2_/ *K*_5_/ *K*_10_	8/5/4	4/5/8
		*F*_2,3_	*K*_3_/ *K*_8_	3/5	5/3
		*F*_2,4_	*K*_1_/ *K*_3_/ *K*_7_	6/2/6	2/6/2

J_3_	1	*F*_3,1_	K3/K9/K10	4/3/4	3/4/3
		*F*_3,2_	K1/K5/K10	3/4/3	4/3/4
		*F*_3,3_	K3/K4/K9	3/6/4	6/3/4

J_4_	1	*F*_4,1_	K2/K4/K7	3/6/4	6/3/4
		*F*_4,2_	K1/K6/K8	6/8/7	8/6/7
		*F*_4,3_	K1/K4/K10	5/8/7	8/5/7
		*F*_4,4_	K5/K6/K9	5/9/3	5/3/9

J_5_	1	*F*_5,1_	K5/K6/K9	2/6/2	6/2/6
		*F*_5,2_	K3/K4	3/7	7/3
		*F*_5,3_	K2/K7/K10	5/4/3	3/4/5
		*F*_5,4_	K1/K3/K5/K8	4/3/4/6	4/6/4/3

J_6_	1	*F*_6,1_	K2/K6	5/6	6/5
		*F*_6,2_	K4/K5/K10	6/4/6	4/6/4
		*F*_6,3_	K2/K6/K9	8/10/6	8/6/10
		*F*_6,4_	K4/K7/K10	10/7/5	5/7/10

J_7_	1	*F*_7,1_	K3/K6	3/8	8/3
		*F*_7,2_	K2/K8	5/5	5/5
		*F*_7,3_	K3/K8	4/7	7/4
		*F*_7,4_	K2/K6/K9	3/7/2	3/2/7
		*F*_7,5_	K2/K4/K8	5/4/6	5/6/4

J_8_	1	*F*_8,1_	K1/K6/K10	5/6/6	6/5/5
		*F*_8,2_	K1/K5/K7	3/3/9	9/9/3
		*F*_8,3_	K3/K6/K9	3/6/4	6/3/4
		*F*_8,4_	K2/K3/K8	5/3/4	3/5/4
		*F*_8,5_	K1/K5/K8	5/3/4	3/5/4
		*F*_8,6_	K1/K9/K10	5/4/4	5/5/4

**Table 2:** Transport cost.

Production line transport cost (10,000 yuan/1,000 pieces)/(day/1,000 pieces)	*K*_1_	*K*_2_	*K*_3_	*K*_4_	*K*_5_	*K*_6_	*K*_7_	*K*_8_	*K*_9_	*K*_10_
*K*_1_	0/0	2/1	1/0.5	2/1	3/1.5	2/1	4/2	4/2	1/0.5	2/1
*K*_2_	3/0.5	0/0	2/1	3/1.5	1/0.5	2/1	2/1	3/1.5	2/1	1/0.5
*K*_3_	2/0.5	3/1.5	0/0	1/0.5	2/2	3/1.5	2/1	2/1	3/1.5	3/1.5
*K*_4_	1/0.5	2/1	2/1	0/0	1/0.5	3/1.5	3/1.5	2/1	3/1.5	2/1
*K*_5_	1/0.5	3/1.5	1/0.5	3/1.5	0/0	2/1	4/2	3./1.5	2/1	3/1.5
*K*_6_	2/0.5	2/1	2/1	4/2	2/1	0/0	3/1.5	3/1.5	4/2	2/1
*K*_7_	3/0.5	3/1.5	3/1.5	3/1.5	2/1	3/1.5	0/0	2/1	3/1.5	2/1
*K*_8_	2/0.5	3/1.5	3/1.5	2/1	2/1	2/1	2/1	0/0	2/1	3/1.5
*K*_9_	3/0.5	2/1	4/2	2/1	3/1.5	4/2	3/1.5	1/0.5	0/0	3/1.5
*R*_10_	1/0.5	2/1	2/1	3/1.5	2/1	5/2.5	2/1	3/1.5	3/1.5	0/0

## Data Availability

The data used to support the findings of this study are available from the corresponding author upon request.
